# ParaHaplo 2.0: a program package for haplotype-estimation and haplotype-based whole-genome association study using parallel computing

**DOI:** 10.1186/1751-0473-5-5

**Published:** 2010-06-04

**Authors:** Kazuharu Misawa, Naoyuki Kamatani

**Affiliations:** 1 Research Program for Computational Science, Research and Development Group for Next-Generation Integrated Living Matter Simulation, and Fusion of Data and Analysis Research and Development Team, RIKEN, 4-6-1 Shirokane-dai, Minato-ku, Tokyo 108-8639, Japan; 2 Laboratory for Statistical Analysis, RIKEN Center for Genomic Medicine, Tokyo, Japan

## Abstract

**Background:**

The use of haplotype-based association tests can improve the power of genome-wide association studies. Since the observed genotypes are unordered pairs of alleles, haplotype phase must be inferred. However, estimating haplotype phase is time consuming. When millions of single-nucleotide polymorphisms (SNPs) are analyzed in genome-wide association study, faster methods for haplotype estimation are required.

**Methods:**

We developed a program package for parallel computation of haplotype estimation. Our program package, ParaHaplo 2.0, is intended for use in workstation clusters using the Intel Message Passing Interface (MPI). We compared the performance of our algorithm to that of the regular permutation test on both Japanese in Tokyo, Japan and Han Chinese in Beijing, China of the HapMap dataset.

**Results:**

Parallel version of ParaHaplo 2.0 can estimate haplotypes 100 times faster than a non-parallel version of the ParaHaplo.

**Conclusion:**

ParaHaplo 2.0 is an invaluable tool for conducting haplotype-based genome-wide association studies (GWAS). The need for fast haplotype estimation using parallel computing will become increasingly important as the data sizes of such projects continue to increase. The executable binaries and program sources of ParaHaplo are available at the following address: http://en.sourceforge.jp/projects/parallelgwas/releases/

## Background

Recent advances in various high-throughput genotyping technologies have allowed us to test allele frequency differences between case and control populations on a genome-wide scale [1]. Genome-wide association studies (GWAS) are used to compare the frequency of alleles or genotypes of a particular variant between cases and controls for a particular disease across a given genome [2-4]. More than a million single-nucleotide polymorphisms (SNPs) are analyzed in SNP-based GWAS. One difficulty faced when conducting SNP-based GWAS is performing corrections for multiple comparisons. Under the assumption that all SNPs are independent, a Bonferroni correction for a P value is usually used to account for multiple tests. When SNP loci are in linkage disequilibrium, Bonferroni corrections are known to be too conservative [5]. As a result, SNP-based GWAS may exclude the truly significant SNPs from analysis [6].

To cope with problems related to multiple comparisons in GWAS, haplotype-based algorithms were developed to correct for multiple comparisons at multiple SNP loci in linkage disequilibrium [5]. A permutation test can also help control inherent problems with multiple testing [6]. The use of haplotype-based association tests can improve the power of GWAS [7,8]. To conduct haplotype-GWAS within a short time period, Misawa and Kamatani [9] developed ParaHaplo 1.0, a set of computer programs for the parallel computation of accurate P values in haplotype-based GWAS by using the MCMC [5] and RAT [6].algorithms.

Despite this, haplotype estimation is still time consuming [10], and therefore, faster methods for haplotype estimation are required. We developed a software package for the parallel computation of haplotype estimation called ParaHaplo 2.0. ParaHaplo 2.0 contains all of the functions of ParaHaplo 1.0 [9]. Additionally, ParaHaplo 2.0 can conduct haplotype estimation by using the PHASE 2.1 [11] and SNPHAP 1.3.1 [12] algorithms. ParaHaplo 2.0, is based on the principle of data parallelism--a programming technique used to split large datasets into smaller ones that can be run in a parallel, concurrent fashion [13]. ParaHaplo 2.0 is intended for use in workstation clusters using the Intel Message Passing Interface (MPI).

Using ParaHaplo 2.0, we estimated haplotypes from the genotype data of the Japanese from Tokyo (JPT), and Han Chinese from Beijing (CHB); these data sets were obtained from the HapMap dataset [14]. Using ParaHaplo 2.0, we compared the speed of haplotype estimation using parallel computation to the number of processors.

## Implementation

### Software overview

ParaHaplo supports the genotype data in the HapMap format [10] as well as the BioBank Japan format [15]. For input, ParaHaplo 2.0 requires a file of haplotype block boundaries. ParaHaplo 2.0 conducts haplotype estimation by using PHASE 2.1 [11] and SNPHAP 1.3.1 [12] algorithms. ParaHaplo 2.0 can also conduct haplotype-based GWAS like version 1.0 [9].

### Parallel computing using MPI methods

ParaHaplo 2.0 is implemented in an MPI-C multithreaded package. The MPI package allows us to construct parallel computing programs on multiprocessors. The genome-wide polymorphism data is broken down into user-defined haplotype blocks, and the MPI Bcast function is used to distribute a single block of haplotype data into each processor. Each processor executes PHASE 2.1 [11] and SNPHAP 1.3.1 [12] algorithms and estimates haplotypes of a single linkage disequilibrium (LD) block. Once the haplotypes of each LD block are completely estimated, the results are compiled into a single genome-wide dataset by using the MPI-Gatherv function. ParaHaplo 2.0 is compatible with OpenMPI 1.2.5 as well as with MPICH 1.2.7p1. Users can compile the source code using a GCC compiler or an Intel C compiler.

## Methods

### Hardware

When computational time was measured, a CentOS PC cluster at RIKEN was used. The program was compiled using an Intel C compiler. Numbers of processing units used were 1, 2, 4, 8, 16, 32, 64, 128, and 256.

### Example data

An example of GWAS is presented here: We used ParaHaplo 2.0 to compare genome-wide genotype data of JPT and CHB from HapMap [14]; the number of individuals therein was 44 and 45, respectively. Haplotype blocks were obtained as LD blocks, using the method outlined by Gabriel *et al*. [16] and by using the Haploview program [17]. The entire genomes of JPT and CHB were divided into 106,149 haplotype blocks by Haploview [17]. PHASE 2.1 does not work with a large number of SNPs [11,18]; therefore, when the number of SNPs in an LD block was greater than 40, we split the block into 40 SNPs.

## Results

### Haplotype Estimation of JPT and CHB

**Figure 1:**
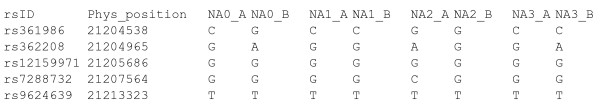
**The result of haplotype phasing**. The first column shows the SNP number. The second column shows the position of SNP in the chromosome. The additional columns display phased haplotypes; each column shows a haplotype. Individuals are separated by a tab; haplotypes are separated by a space.

Figure 1 shows the result of haplotype phasing. The SNP number, the position of the SNP in the chromosome, and haplotype data are displayed in each line; the rest are phased haplotypes. Each column displays a haplotype. Individuals are separated by a tab; haplotypes are separated by a space. The data format is identical to the results from ParaHaplo 1.0 [9].

### Calculation Time

The speedup ratio is the ratio of the computation time of a single processor to that of multiple processors. Table 1 shows the elapsed times and the speedups associated with the use of ParaHaplo 2.0 using the genotype data of chromosome 22 for haplotype estimation. In table 2, the calculation time decreased as the number of processors increased. When 256 processors were used, ParaHaplo was 100 times faster than the non-parallel program.

**Table 1:** Elapsed times and speedups obtained with ParaHaplo applied on the HapMap 3 JPT and CHB data of chromosome 22.

Elapsed times and speedups obtained with ParaHaplo on the phasing process							
**Number of Processing Units**	**Calculation Time**						**Speed Ratio **^a^

1	9	h	56	m	54	s	1
2	4	h	56	m	13	s	2
4	2	h	26	m	40	s	4
8	1	h	21	m	39	s	7
16			39	m	2	s	15
32			21	m	5	s	28
64			11	m	49	s	50
128			7	m	4	s	85
256			5	m	32	s	108

^a^Ratio of computation time of a single processor to computation time of multiple processors

## Discussion

We developed ParaHaplo 2.0, a set of computer programs, for the parallel computation of haplotype estimation as well as for accurate P values in haplotype-based GWAS. ParaHaplo is intended for use in workstation clusters using the Intel MPI. By using ParaHaplo, we conducted haplotype estimation of the genotype data of JPT and CHB from the HapMap dataset [14].

### Parallel Computation of Haplotype-based GWAS

The results showed that the parallel computing ability of ParaHaplo 2.0 for haplotype estimation was 100 times faster than non-parallel version of ParaHaplo 2.0. In this study, we used a total of 89 JPT and CHB individuals whose genotypes had been determined during the HapMap project [14]. When a single processor was used, haplotype estimation for chromosome 22 took more than 9 h; if 9,000 individuals were to be analyzed under the same conditions, it would take approximately 1 month. However, if ParaHaplo 2.0 was used on a workstation with 256 processors, the same analysis would take approximately 9 h.

Algorisms for faster haplotype estimation, such as FastPHASE [19] and GERBIL [20], have been developed. However, we chose PHASE 2.1 [11] because it outperforms these methods in accuracy of estimating haplotypes of these methods [19].

Even when 256 processors were used, the speedup ratio was only 116 because of the variations in the LD block size. Since ParaHaplo is based on data parallelism, the computation times of each haplotype estimation was approximately proportional to the number of SNPs within the LD block [5,6]; therefore, we believe that a large LD block may becomes a computational bottleneck. PHASE 2.1 [11] in ParaHaplo 2.0 does not work for a large number of SNPs, when the number of SNPs in a haplotype block is greater than 40. Most of SNPs in a large LD block are in strong LD so that we must choose smaller number of tag SNPs in phase estimation to estimate haplotypes by using PHASE 2.1 [11]. Or, we can use SNPHAP 1.3.1 [12] in ParaHaplo 2.0.

## Conclusion

The results indicated that when the number of processors is sufficient, the parallel computing abilities of ParaHaplo were 100 times faster than those of non-parallel programs. There are more than a million SNPs for which accurate and complete genotypes have been obtained [15], more than ten thousands of people are now being genotyped [21]. The need for fast haplotype estimation using parallel computing will become increasingly important as the data sizes of such projects continue to increase.

## Availability and Requirements

• **Project name**: ParaHaplo 2.0

• **Project home page**:. http://sourceforge.jp/projects/parallelgwas/releases/46982

• **Operating systems**: Platform independent

• **Programming language**: Java and C

• **Other requirements**: OpenMPI version 1.2.5, or MPICH version 1.2.7p1

• **License**: MIT license

• **Any restrictions to use by non-academics**: License required

## Abbreviations

RAT: Rapid Association Test; SPT: Standard Permutation Test; MCMC: Markov-chain Monte Carlo; JPT: Japanese Tokyo; CHB: Han Chinese Beijing.

## Competing interests

The authors declare that they have no competing interests.

## Authors' contributions

KM wrote the software and the manuscript, and NK supervised the project. Both authors read and approved the final manuscript.

## References

[B1] Hirschhorn JN Daly MJ Genome-wide association studies for common diseases and complex traits Nat Rev Genet 2005 6 95 108 10.1038/nrg1521 15716906

[B2] Ozaki K Ohnishi Y Iida A Sekine A Yamada R Tsunoda T Sato H Hori M Nakamura Y Tanaka T Functional SNPs in the lymphotoxin-alpha gene that are associated with susceptibility to myocardial infarction Nat Genet 2002 32 650 654 10.1038/ng1047 12426569

[B3] Onouchi Y Gunji T Burns JC Shimizu C Newburger JW Yashiro M Nakamura Y Yanagawa H Wakui K Fukushima Y Kishi F Hamamoto K Terai M Sato Y Ouchi K Saji T Nariai A Kaburagi Y Yoshikawa T Suzuki K Tanaka T Nagai T Cho H Fujino A Sekine A Nakamichi R Tsunoda T Kawasaki T Hata A ITPKC functional polymorphism associated with Kawasaki disease susceptibility and formation of coronary artery aneurysms Nat Genet 2008 40 35 42 10.1038/ng.2007.59 18084290 PMC2876982

[B4] Tokuhiro S Yamada R Chang X Suzuki A Kochi Y Sawada T Suzuki M Nagasaki M Ohtsuki M Ono M Furukawa H Nagashima M Yoshino S Mabuchi A Sekine A Saito S Takahashi A Tsunoda T Nakamura Y Yamamoto K An intronic SNP in a RUNX1 binding site of SLC22A4, encoding an organic cation transporter, is associated with rheumatoid arthritis Nat Genet 2003 35 341 348 10.1038/ng1267 14608356

[B5] Misawa K Fujii S Yamazaki T Takahashi A Takasaki J Yanagisawa M Ohnishi Y Nakamura Y Kamatani N New correction algorithms for multiple comparisons in case-control multilocus association studies based on haplotypes and diplotype configurations J Hum Genet 2008 53 789 801 10.1007/s10038-008-0312-0 18651098

[B6] Kimmel G Shamir R A fast method for computing high-significance disease association in large population-based studies Am J Hum Genet 2006 79 481 492 10.1086/507317 16909386 PMC1559554

[B7] Schaid DJ Evaluating associations of haplotypes with traits Genet Epidemiol 2004 27 348 364 10.1002/gepi.20037 15543638

[B8] Browning BL Browning SR Efficient multilocus association testing for whole genome association studies using localized haplotype clustering Genet Epidemiol 2007 31 365 375 10.1002/gepi.20216 17326099

[B9] Misawa K Kamatani N ParaHaplo: A program package for haplotype-based whole-genome association study using parallel computing Source Code Biol Med 2009 4 7 10.1186/1751-0473-4-7 19845960 PMC2774321

[B10] Marchini J Cutler D Patterson N Stephens M Eskin E Halperin E Lin S Qin ZS Munro HM Abecasis GR Donnelly P A comparison of phasing algorithms for trios and unrelated individuals Am J Hum Genet 2006 78 437 450 10.1086/500808 16465620 PMC1380287

[B11] Mardanov AV Ravin NV Kuznetsov BB Samigullin TH Antonov AS Kolganova TV Skyabin KG Complete Sequence of the Duckweed (Lemna minor) Chloroplast Genome: Structural Organization and Phylogenetic Relationships to Other Angiosperms J Mol Evol 2008 66 6 555 64 10.1007/s00239-008-9091-7 18463914

[B12] SNPHAP - A program for estimating frequencies of large haplotypes of SNPs http://www-gene.cimr.cam.ac.uk/clayton/software/

[B13] Culler DE Gupta A Singh JP Parallel Computer Architecture: A Hardware/Software Approach 1997 San Francisco, CA: Morgan Kaufmann Publishers

[B14] The International HapMap Consortium The International HapMap Project Nature 2003 426 789 796 10.1038/nature02168 14685227

[B15] Nakamura Y The BioBank Japan Project Clin Adv Hematol Oncol 2007 5 696 697 17982410

[B16] Gabriel SB Schaffner SF Nguyen H Moore JM Roy J Blumenstiel B Higgins J DeFelice M Lochner A Faggart M Liu-Cordero SN Rotimi C Adeyemo A Cooper R Ward R Lander ES Daly MJ Altshuler D The structure of haplotype blocks in the human genome Science 2002 296 2225 2229 10.1126/science.1069424 12029063

[B17] Barrett JC Fry B Maller J Daly MJ Haploview: analysis and visualization of LD and haplotype maps Bioinformatics 2005 21 263 265 10.1093/bioinformatics/bth457 15297300

[B18] Browning SR Missing data imputation and haplotype phase inference for genome-wide association studies Hum Genet 2008 124 439 450 10.1007/s00439-008-0568-7 18850115 PMC2731769

[B19] Scheet P Stephens M A fast and flexible statistical model for large-scale population genotype data: applications to inferring missing genotypes and haplotypic phase Am J Hum Genet 2006 78 629 644 10.1086/502802 16532393 PMC1424677

[B20] Kimmel G Shamir R GERBIL: Genotype resolution and block identification using likelihood Proc Natl Acad Sci USA 2005 102 158 162 10.1073/pnas.0404730102 15615859 PMC544046

[B21] Kamatani Y Matsuda K Okada Y Kubo M Hosono N Daigo Y Nakamura Y Kamatani N Genome-wide association study of hematological and biochemical traits in a Japanese population Nat Genet 2010 42 210 215 10.1038/ng.531 20139978

